# Book review: Chasing After Chimpanzees: The Making of a Primatologist

**DOI:** 10.5194/pb-9-5-2022

**Published:** 2022-05-16

**Authors:** Kevin D. Hunt

**Affiliations:** Department of Anthropology, Indiana University, Bloomington, Indiana, USA​​​​​​​

*McGrew, W. C.: Chasing After Chimpanzees: The Making of a Primatologist, Mereo Press, Cirecester, UK, 275 pp., ISBN 978-1-86151-582-7, USD 14.00 (softcover), 2021.*

If you know the name of only one chimpanzee scholar, that name is Jane Goodall. But if you know half a dozen such names, you know the name Bill McGrew.

Race horse owner, Hibs fan and beer enthusiast, McGrew's life has been packed with adventure – chimpanzee research aside – and in this volume he recalls that life with great humor and gusto.

I picked up *Chasing After Chimpanzees* expecting to learn of the genesis of some of McGrew's signature contributions to animal behavior. But I also hoped – I confess – to read a juicy anecdote or two revealing youthful indiscretions of one or two of my now-senior colleagues. There is a little of the former in *Chasing After Chimpanzees* but none of the latter. Instead, McGrew recounts the more private aspects of his life in 110 brief, lucid, absorbing vignettes. It makes for a rousing page-turner.

In childhood McGrew survived a direct tornado strike, and he prospected for fossil mammals in the Arbuckle Mountains. As a teen he provisioned iguanas by rolling food balls into their cage, snatched glimpses of a famous chimpanzee breeding colony and journeyed to the Bahamas as a one-man expedition stalking the elusive curly tailed lizard. As a young man he dined with J. R. R. Tolkien, won (as a teammate of Bill Bradley) an English national basketball championship (earning a double Oxford Blue along the way), guarded Pat Riley in international competition, emigrated to Scotland and marched in the largest Vietnam war protest in Britain. In full adulthood he captured a 5 ft *Boa constrictor* with his bare hands, ran five marathons, coached the University of Stirling women's basketball team to the Scottish Universities' championship game, survived incarceration as an illegal alien in Côte D'Ivoire, lent his name to a new species of termite (*Proboscitermes mcgrewi*), drove 6000+ miles from Scotland to Senegal across the Sahara and flew in an airplane piloted (however briefly) by a chimpanzee. These are but a few of his life adventures.

While of less interest to a layperson, McGrew's briefly reviewed professional achievements are, to an academic, every bit as riveting as his private life. He published his first article as a teenager: on catfish prey if you can believe it (McGrew, 1963). If you are a primatologist, you have never even heard of his first book: *An Ethological Study of Children's Behavior*, a volume reviewed in *Nature* and *Science* and cited an extraordinary 757 times (McGrew, 1972). In fact, more than half of his first dozen publications concerned child social development. His primatology career began only slowly, initially when he studied captive chimpanzees at the Delta Regional Primate Research in 1972; he drifted further into primatology with an almost accidentally acquired Stanford postdoc; he was soon a full-blown primatologist. As an author, coauthor and editor, he has published 180 articles, 56 book chapters, 8 books and nearly 150 book reviews. He has published 44 articles with Linda Marchant alone, a career output for many academics. His ∼ 25 000 citations, as per Google Scholar, place him in the citation stratosphere with Goodall herself.

In our personal interactions, he has astonished me now and then with his knowledge of this or that abstruse area of scholarship. No wonder; in high school he became proficient in entomology, herpetology and botany. During his undergraduate years he rubbed elbows with half a score of A-list primatologists, won a Rhodes Scholarship and then began doctoral studies with Niko Tinbergen at Oxford. He has visited 60 countries in the course of his career and engaged in chimpanzee research at nine separate chimpanzee field sites – and visited a further four. His research extends far beyond chimpanzees: he has studied 18 other species of primate. He was press-ganged into directing a captive callithrichid colony at the University of Stirling and dramatically improved their lot, guiding the colony to the highest infant survival rate in the world and coinventing an artificial gum tree along the way.

At many points in McGrew's narrative my mind turned toward career-oriented discussions I have had with my current and former students. How does one get his or her first position? How to get tenure? What does it take to rise to prominence in one's field? McGrew's career is an object lesson: take seriously advice from mentors; pounce on opportunities; accept that luck will play a part in your career; pursue a fulfilling life outside academia; remind yourself that even stars suffer disappointments; and (if you wish to attain real distinction) churn out eight noteworthy publications a year.

## References

[bib1.bib1] McGrew WC (1963). Channel catfish feeding on diamond-backed water snakes. Copeia.

[bib1.bib2] McGrew WC (1972). An Ethological Study of Children's Behavior.

